# The emergence of telemedicine in a low-middle-income country: challenges and opportunities

**DOI:** 10.3332/ecancer.2024.1679

**Published:** 2024-03-01

**Authors:** Kofi Adesi Kyei, George Nakoja Onajah, Joseph Daniels

**Affiliations:** 1 Department of Radiography, University of Ghana, Legon, Accra, Ghana; 2 Accra Business School, Leaders Factory, Spintex, Accra, Ghana; 3 National Radiotherapy, Oncology and Nuclear Medicine Centre, Korle Bu Teaching Hospital, P.O. Box KB 369, Korle Bu, Accra, Ghana; a https://orcid.org/0000-0003-3485-5368; b https://orcid.org/0000-0002-1466-150X

**Keywords:** telemedicine, healthcare in Ghana, health infrastructure, remote consultation, internet connectivity, remote cancer care

## Abstract

The quality of cancer care delivery varies across different regions of Ghana, highlighting the need for improved access to quality healthcare services. Telemedicine has emerged as a promising solution to address this disparity, as it can reduce costs and improve access to healthcare services for cancer patients in remote areas. Despite the widely reported benefits of telemedicine, its adoption in low-resource settings has been slow due to several challenges. This study explores strategies for incorporating telemedicine into the current healthcare system in Ghana for the benefit of all patients especially those diagnosed with cancer. The study also highlights the current challenges and opportunities associated with the implementation and utilisation of telemedicine in Ghana. This research was a cross-sectional study conducted in Accra, Ghana that adopted a mixed-methods approach. Participants were selected through multi-stage probability sampling. Quantitative data were collected via a survey whereas qualitative data were obtained by means of in-depth interviews and focus group discussions among healthcare professionals, patients and key stakeholders in the telemedicine industry. The Statistical Program for the Social Sciences (version 21) was used to assemble, analyse and display the research data. The major challenges discussed centered on high initial investment costs, privacy and security concerns, poor internet connectivity, insufficient infrastructure and training of healthcare providers as well as the resistance to change among healthcare professionals. The study contributes to the understanding of telemedicine adoption in Ghana with findings underscoring the potential to address healthcare challenges while highlighting the need to overcome implementation obstacles. The study findings also provide valuable insights for policymakers, healthcare institutions and stakeholders to enhance telemedicine adoption in Ghana.

## Introduction

Access to quality healthcare remains a significant challenge in many low-middle-income countries, including Ghana, especially in rural areas [1]. Limited funding and a perennial shortage of trained healthcare professionals disproportionately affect rural areas where the doctor and nurse-to-population ratios are very low. The quality of cancer care delivery varies across different regions of the country, highlighting the need for improved access to quality healthcare services. Ghana adopted the Community Health Planning and Services approach in 1994 as a strategy to provide primary healthcare services on a community basis to increase access to basic healthcare services in deprived and underprivileged areas of the country [2, 3].

Telemedicine has also emerged as a promising solution to address this disparity, as it can reduce costs and improve access to healthcare services for patients especially those with cancer in remote areas of resource-limited settings [4]. Telemedicine is an increasingly popular concept in both developed and developing countries. Several terms are used interchangeably with telemedicine, including telehealth, telecare, telenursing and telematics [5]. Telemedicine involves the use of telecommunications technology to provide medical information and services, allowing for the electronic transmission of medical data for diagnosis, therapy and education. Telemedicine services are commonly categorised into four main types: tele-consultation, tele-education, tele-monitoring and tele-surgery [5]. Telemedicine systems typically include communication devices with software for sharing information and a secure network channel to ensure the confidentiality and privacy of patient information [6].

Telemedicine has been used in high-income countries to address health disparities and provide solutions to the shortage of healthcare professionals, especially in underserved communities [7]. Given the increasing population of aged people and the prevalence of age-related chronic diseases worldwide, telemedicine also provides access to healthcare among the aged and reduces frequent visits to healthcare facilities [8]. The COVID-19 pandemic has accentuated the enormous potential of telemedicine to alleviate the immense burden placed on health professionals and healthcare facilities with in-person patient services [9]. Studies in high-income countries have demonstrated that the use of telemedicine in the delivery of home healthcare services results in reduced mortality, better medication compliance and improved safety [10].

A study involving health workers at six purposively selected health facilities in Ghana reported a growing acceptance and readiness among health professionals to adopt telemedicine in Ghana [11]. Another study examining the use of telemedicine at two large medical facilities in Ghana demonstrated a heightened interest among physicians and hospital administrators in the utilisation of telemedicine as an adjunct to in-person medical care [12].

Despite the widely reported benefits of telemedicine, its adoption in low-resource settings has been slow due to several challenges including inadequate investment in telecommunications infrastructure, limited internet connectivity and the need for regulatory frameworks to ensure the confidentiality and privacy of patient information [13]. A recent study identified financial, technological and organisational constraints as the major impediments to the widespread use of telemedicine in Ghana. The purpose of this study was to explore the major challenges and opportunities associated with the emergence of telemedicine in a resource-limited country.

## Methods

This research was a cross-sectional study conducted in the Greater Accra region of Ghana that adopted a mixed-methods approach. The study population comprised individuals who have a stake in the telemedicine industry in general and how it relates to cancer care in particular. Policymakers who have been involved in the implementation of telemedicine policies and regulations in the Greater Accra region were included in the study population. Based on Yamane’s approach [14], the appropriate sample size for this study was determined to be 109 participants. A purposive sampling technique and multi-stage probability sampling method were used to select the participants for this study. In the first stage of the sampling process, the study area was divided into five groups based on the five different administrative districts of the Greater Accra region. A random sample of districts was then selected to represent the entire region. In the second stage, healthcare facilities including hospitals, clinics, medical and telemedicine centres within the selected districts were identified for participant recruitment. In the third stage, patients who had previously used telemedicine services were identified and sampled from each selected healthcare facility. Both quantitative and qualitative data were collected.

The quantitative data was collected via a survey of carefully selected healthcare professionals whereas the qualitative data was obtained by means of in-depth interviews and focus group discussions among healthcare professionals, patients and key stakeholders in the telemedicine industry. The focus group discussions focused on four key areas: the adoption of telemedicine, infrastructure, regulations and patients’ acceptance of telemedicine as a safe and effective approach to primary healthcare. A structured questionnaire was designed to elicit information about the availability, current extent of utilisation, challenges faced by healthcare providers and the potential opportunities for the development of telemedicine. The reliability of the research instrument was determined to be acceptable based on a coefficient of Cronbach’s alpha of 0.74. and the face validity was confirmed by two independent reviewers with expertise in telemedicine in Ghana. Pilot testing of the research instruments was also done to ensure that the questionnaires and interview questions were appropriate, easy to understand and suitable for eliciting the required data for the study. For this purpose, the questionnaire was administered to a sample of 15 healthcare providers, including doctors, nurses and other healthcare professionals who have experience with telemedicine or have the potential to use telemedicine in their practice. Version 21 of the Statistical Program for the Social Sciences was used to assemble, analyse and display participant data. Descriptive statistics including frequency distribution, percentages, medians and means were used to summarise the quantitative data. The study commenced after obtaining institutional review board approval from the Accra Business School (reference number: ABS-KN-MB22332/23/132). Written informed consent was obtained from all participants prior to their recruitment in this study. The confidentiality and privacy of the respondents were maintained during the conduct of this study. Patients’ identifying information were removed from all extracted medical records. The study adhered to ethical principles and standards, including the declaration of Helsinki.

## Results

The response rate was 91.7%, with a total number of 100 participants responding to the survey. The male-to-female sex ratio of the study participants was 45% versus 55%. In all, 20% of the respondents were <25 years and 25% > 40 years whereas 30% were between 26- and 30-years as shown in Table 1 which is a summary of the baseline characteristics of the study participants. The highest level of education of half (50%) of the respondents was either a diploma or bachelor’s degree. In all, 40% of the participants were health workers and 25% were health administrators whereas 30% were patients familiar with telemedicine.

Table 2 summarises the response of the participants to items on the questionnaire regarding the benefits of telemedicine. In all 25% of the participants strongly agreed whereas 5% strongly disagreed with the following potential benefits of telemedicine: ‘the use of telemedicine accelerates the clinical decision-making process’, ‘accessing patients’ previous medical records is easier with telemedicine’ and ‘telemedicine has the potential to enhance the overall quality of patient care’. The lowest mean scores of 3.0 (SD 0.4) and 3.3 (SD 0.5) were recorded respectively for the statements ‘transitioning from face-to-face healthcare services to telemedicine has interfered with my overall performance’ and ‘I prefer the use of telemedicine to face-t-face consultations with patients’.

The highest means scores of 3.7 (SD 0.7) and 3.8 (SD 0.7) were recorded for these items on the questionnaire: ‘telemedicine training was effectively organised, with competent trainers and appropriate training resources’ and ‘telemedicine provides tools that make it possible to perform all required functions’. With a mean score of 2.7 (SD 0.2), 25% of the respondents disagreed that ‘there is significant distrust of telemedicine’ as illustrated in Table 3 which summarises the response of the participants to items on the questionnaire regarding the challenges associated with the implementation of telemedicine. Also, 30% of the respondents strongly agreed that ‘telemedicine provides tools that make it possible to perform all required functions’.

Table 4 is a summary of the key points from the focus group discussions which were centered around the broad themes of ‘the challenges associated with the implementation and utilisation of telemedicine’, ‘potential strategies to tackle the challenges associated with the implementation and utilisation of telemedicine’ and ‘opportunities for promoting and expanding accessibility, acceptability and utilisation of telemedicine’. The major challenges discussed centered around high initial investment costs, privacy and security concerns, poor internet connectivity, insufficient infrastructure and training of healthcare providers as well as the resistance to change among healthcare professionals. The strategies to tackle these challenges include the exploration of cost-effective telemedicine solutions for deprived areas and allocation of sufficient resources for infrastructure development as well as the comprehensive training of healthcare professionals and awareness creation campaigns.

## Discussion

The potential benefits of telemedicine in Ghana are endless. By leveraging information and communication technologies, telemedicine can provide remote access to healthcare services, including diagnosis, treatment and evaluation of patients. This can significantly reduce the cost associated with providing quality healthcare, particularly in regions where constructing and staffing new facilities is not feasible [15]. The appropriate use of modern information technology has the potential to improve clinical care and public health, as well as medical education, administration and research. It can improve access to healthcare services, enhance the quality of healthcare delivery and mitigate the impact of the global shortage of healthcare professionals [16]. The emergence of telemedicine in Ghana is associated with multiple challenges and opportunities for development and improvement.

## Challenges

The implementation and utilisation of telemedicine in Ghana is fraught with a lot of challenges. Limited internet connectivity is a prominent obstacle that greatly hinders the widespread adoption and utilisation of telemedicine services in resource-limited settings that are typically characterised by poor access to high-speed internet services. It is also a well-known barrier in many rural or underserved urban areas. Robust telecommunication infrastructure is indispensable for the efficient and reliable use of telemedicine services [15]. Even though telecommunication network coverage has significantly expanded in Ghana over the past decade, there are still pockets of densely inhabited areas where internet and phone service coverage are either absent, poor or unstable. High-speed internet services are readily available in many urban centres but unfortunately at a cost that is not affordable for many. The inability of poor rural dwellers to afford expensive high-speed internet services is also a disincentive for telecommunication companies to invest in extending the range of these services to such areas. There is an urgent need for infrastructure development to bridge the digital divide especially between high and low-income countries to ensure equitable access to telemedicine services [17]. Inadequate investment in telecommunications infrastructure, particularly in regions affected by wars and civil unrest, has also hindered the implementation of telemedicine in Africa. This has made it difficult to provide essential services in rural areas where infrastructure requirements are high and internet connectivity is often limited [18].

Inherent resistance to change among healthcare professionals and patients alike is a common challenge in the adoption of new technologies that impacts the utilisation of telemedicine negatively. The transition from traditional face-to-face encounters to telemedicine oftentimes disrupts established practices and requires healthcare providers as well as end users to adapt to the changing circumstances. Users of healthcare services are bound to have reservations about the effectiveness, quality and safety of telemedicine during this transition. Healthcare professionals must be adequately trained and equipped with the necessary skills and knowledge to navigate telemedicine platforms effectively [19]. Comprehensive training programs and awareness creation campaigns are crucial to overcoming this resistance and ensuring the effective assimilation of telemedicine [20].

Privacy and security concerns were also identified as major barriers impeding the wholesale adoption of telemedicine. The respondents of the study expressed the urgent need for robust encryption and data protection measures to safeguard patient information. The importance of maintaining patient confidentiality and implementing secure telemedicine platforms has been previously documented [21].

## Opportunities

Despite the enumerated challenges associated with telemedicine, there are a lot of potential opportunities for expanding its accessibility, acceptability and utilisation in low resource settings such as Ghana. The results of this study gives credence to the fact that telemedicine accelerates the clinical decision-making process, facilitates access to patients’ previous medical records and can enhance the overall quality of patient care. There is ample published evidence supportive of the positive role of telemedicine in improving efficient delivery of primary healthcare and patient outcomes [22].

Telemedicine offers various services including teleconsultation, online visits and home visits, providing immediate medical care and advice to patients. These benefits of telemedicine have the potential to revolutionise healthcare delivery in Ghana. For example, tele-monitoring, also known as home telehealth or remote monitoring which involves the use of various technological devices to monitor a patient’s health, vital signs and clinical indicators from a distance can solve the challenge posed by long distances and impassable roads between healthcare facilities and remote regions. For cancer survivors who are undergoing pot-treatment surveillance with periodic assessment of tumour markers, the need to travel to the cancer treatment centre for reviews can be reduced if the opportunity to remotely assess their test results was created. This would allow carer givers to selectively schedule in-person appointments for patients with abnormal results or those who require physical examination.

Telemedicine can provide a smart and effective way to reduce unnecessary patient visits to the hospital. There are a lot of instances when patients report to the hospital with concerns about their health that are in keeping with normal physiological processes and do not require any pharmacological intervention. These patients are usually reassured and instructed to report again if there are any new symptoms. Arguably the amount of in-person time spent by the clinician in dealing with this situation would be better used attending to a patient with a condition that requires immediate or urgent intervention. Telemedicine can therefore serve as a form of electronic triaging system with the purpose of determining who must be seen by the doctor in-person and who can be attended to virtually.

The respondents of the study expressed varying levels of agreement regarding their preference for telemedicine consultations over face-to-face encounters. While some participants preferred telemedicine, others expressed a preference for in-person consultations, citing a greater sense of control and connection. Whilst some users of telemedicine services prefer the convenience and flexibility of telemedicine, others on the other hand value the personal connection and control offered by in-person consultations. These findings indicate that individual preferences and comfort levels play an important role in the adoption of telemedicine. Telemedicine should be customised as much as possible to accommodate individual patient preferences concerning the appropriate use of audio-visual tools and dedicated communication gadgets. It is noteworthy that most of the respondents expressed confidence in the inherent ability of telemedicine services to maintain patient confidentiality, data protection and regulation of access to private patient medical information. These perceptions of security are crucial for building trust among healthcare providers and patients [23].

Continuous updates and improvements to the user interface of telemedicine services should be provided as this will ultimately enhance user-friendliness and improve user experience with telemedicine technological tools. Integrating artificial intelligence and machine learning algorithms to enhance diagnostic capabilities can optimise the potential of technology to augment healthcare decision-making [24]. Standardising protocols and guidelines for telemedicine consultations is another important initiative that can enhance the effectiveness of telemedicine. Establishing clear guidelines can ensure the practice of evidence-based contactless medicine, the promotion of quality remote healthcare and address patients’ concerns regarding the reliability and trustworthiness of medical care delivered via telemedicine. Regular evaluations and feedback sessions among both known and potential users of telemedicine can facilitate the identification of core areas for improvement. Based on the concept of continuous quality improvement, this strategy can help to detect and address shortcomings in the telemedicine industry.

Collaboration with regulatory bodies to ensure compliance with legal and ethical standards is crucial for the successful implementation of telemedicine. Regulatory frameworks play a vital role in ensuring patient safety, and privacy, and maintaining the quality of care provided through telemedicine [6]. Promoting interoperability between different telemedicine platforms is essential for seamless communication and data exchange which can enhance care coordination, continuity and patient satisfaction. This can also simplify patient information sharing between different healthcare providers.

Tele-consultation refers to patient-clinician interactions which take place on a virtual platform with the participants being in different geographical locations. Sometimes patients’ medical needs do not require that they are seen physically be their clinicians. Long hospital queues can be avoided if such patients were selectively taken care of via telemedicine. This approach could also make it possible to extend consultation hours for clinicians and make it feasible to work from home. It would also save clinicians the time required to travel between one health facility and the other if they were to see patients from these facilities remotely. Tele-radiology involves sending a patient’s radiographs and other imaging data securely to radiologists for evaluation. This service would enable a single radiologist to require the imaging data of several patients in remote areas where there is no radiologist at post.

## Limitations

The study was conducted in the capital city of Ghana which is relatively more affluent and developed than many other cities in the country. It also has the largest concentration of healthcare facilities and telecommunications infrastructure in the country. Hence there might be additional challenges to the emergence of telemedicine peculiar to remote areas in Ghana that were not captured in this study. Caution should be exercised in extrapolating the findings of this study to other cities in the subregion where cultural differences and stereotypes may render telemedicine undesirable to many. Exploration of cultural beliefs, societal attitudes towards healthcare, government policies and regulations were beyond the scope of this study even though these factors also influence the development of the telemedicine industry.

## Conclusion

Telemedicine provides several advantages, including quicker clinical decision-making, improved access to medical records and potential enhancement of the quality of patient care. However, in resource-limited settings like Ghana, its implementation is associated with a myriad of challenges such as infrastructural limitations, insufficient training for healthcare providers, high initial investment costs and resistance to change among both healthcare professionals and patients. Despite these hurdles, there are significant opportunities for expanding telemedicine in such settings, with potential to improve accessibility, acceptability and utilisation of healthcare services.

## Conflicts of interest

The authors declare no competing interest.

## Funding

This study did not receive any specific funding support from funding agencies in the public, commercial, or not-for-profit sectors.

## Data availability

The data used to support the findings of this study are available from the corresponding author upon reasonable request.

## Figures and Tables

**Table 1.** Baseline characteristics of the respondents (*n* = 100).

Variable	Number	Proportion (%)
Gender		
Male	45	45
Female	55	55
Age range (years)		
<25	20	20
26–30	30	30
31–35	15	15
36–40	10	10
>40	25	25
Highest level of education		
Diploma/bachelor’s degree	50	50
Masters or doctoral degree	35	35
Other	15	15
Category of respondents		
Healthcare worker	40	40
Health Administrator	25	25
Patient	30	30
Other	5	5
Years of experience of healthcare workers (*n* = 40)		
<5 years	25	25
5–10 years	10	10
>10 years	5	5

**Table 2.** Participants’ response regarding the benefits of telemedicine.

Questionnaire items	Participants’ response					
	Strongly disagree 1	Disagree 2	Neutral 3	Agree 4	Strongly agree 5	Mean score/(SD)
1. The use of telemedicine accelerates the clinical decision-making process.	5	15	10	45	25	3.7 (0.7)
2. Accessing patients' previous medical records is easier with telemedicine.	5	10	15	45	25	3.8 (0.7)
3. Telemedicine has the potential to enhance the overall quality of patient care.	5	10	10	50	25	3.8 (0.8)
4. Transitioning from face-to-face healthcare services to telemedicine has interfered with my overall performance.	15	20	30	25	10	3.0 (0.4)
5. I feel much more in control using face-to-face encounters with patients than using telemedicine.	5	10	15	45	25	3.8 (0.7)
6. Implementing a telemedicine system improves the confidentiality of patients’ records.	5	10	20	45	20	3.7 (0.7)
7. I prefer the use of telemedicine to face-to-face consultations with patients.	10	15	25	35	15	3.3 (0.5)

**Table 3.** Challenges associated with the implementation of telemedicine.

Questionnaire items	Strongly disagree	Disagree	Neutral	Agree	Strongly agree	Mean (SD)
1. Telemedicine training was effectively organised, with competent trainers and appropriate training resources.	5	10	20	40	25	3.7 (0.7)
2. The adoption of telemedicine places additional workload on clinicians and other users.	10	20	30	30	10	3.1 (0.4)
3. There is significant distrust of telemedicine	25	20	30	15	10	2.7 (0.2)
4. Telemedicine provides tools that make it possible to perform all required functions.	5	10	15	40	30	3.8 (0.7)
5. Telemedicine requires more time to complete the same tasks in face-to-face consultations.	15	20	25	25	15	3.1 (0.3)

**Table 4.** Summary of key points from focus group discussions.

Theme	Key points
1. The challenges associated with the implementation and utilisation of telemedicine	1. Resistance to change among healthcare professionals
	2. Insufficient infrastructure and technical support
	3. Limited internet connectivity in certain areas
	4. Lack of training for healthcare providers
	5. High initial investment costs
	6. Privacy and security concerns
2. Potential strategies to tackle the challenges associated with the implementation and utilisation of telemedicine	1. Conducting awareness campaigns to promote acceptance and understanding among healthcare professionals and patients
	2. Allocating sufficient resources for infrastructure development and technical support
	3. Expanding access to high-speed internet connectivity in rural areas
	4. Providing comprehensive training programs for healthcare providers
	5. Exploring cost-effective telemedicine solutions for resource-limited settings
	6. Addressing privacy and security concerns through robust encryption and data protection measures
3. Opportunities for promoting and expanding accessibility, acceptability and utilisation of telemedicine.	1. Continuous update of the user interface for better usability
	2. Integrating artificial intelligence and machine learning algorithms to enhance diagnostic capabilities
	3. Development of protocols and guidelines for telemedicine consultations
	4. Regular evaluation to identify areas for improvement
	5. Collaboration with regulatory bodies to ensure compliance with legal and ethical standards
	6. Promotion of interoperability between different telemedicine platforms to facilitate seamless communication and data exchange
